# Researching arts, culture, migration and change: a multi (trans)disciplinary challenge for international migration studies

**DOI:** 10.1186/s40878-022-00281-5

**Published:** 2022-02-01

**Authors:** Marco Martiniello

**Affiliations:** FRS-FNRS and University of Liège, Liège, Belgium

**Keywords:** Migration, Arts, Culture, Methodology

## Abstract

The paper first discusses why it is important to research the relations between migration, arts, and cultures. Second, it discusses the most promising methodological options to do it fruitfully. It concludes by claiming that the additional value of such investigations is both to allow a more comprehensive understanding of the migration process, and to move away from the victimization of migrants “rehumanize” them.

## Introduction

The links between arts, culture and migration have long been studied in cultural studies and the Humanities. This research area, however, has been relatively neglected in the growing international field of migration studies in Europe. The field has historically been dominated by demography, sociology, and political science (Martiniello, 2015). The reasons for this neglect reflect the dominant approach to migration and migrants since the end of WWII. First, for decades, migration was essentially perceived as a conjunctural economic phenomenon. Migrants were considered mainly as workers and as forces of production in a globalizing economy. As Sayad (1991) reminds us, the legitimacy of their presence was exclusively based on work, on their status as workers and on their direct contribution to the economy. In that context, their cultural activities and participation were almost totally ignored. The only function of migrants was to work and not to be consumers or producers of arts and culture. Furthermore, as most migrants only enjoyed a low level of formal education and were coming from deprived areas of the south (Europe and beyond), the idea that they could have artistic tastes or that they could be artists, in addition to being manual workers, was not taken seriously. Second, the lack of interest in migrants’ artistic and cultural life also mirrored a more general neglect of arts and culture in general, both in policy debates and in social sciences. If we take the example of sociology, sociology of arts and culture is often considered a minor and marginal subdiscipline that deals with trivial topics.

With the progressive recognition of the fact that migration was not conjunctural but structural, the issue of integration emerged, in most countries of immigration, both as a policy and as an academic issue. Culture, in an anthropological sense, became a contested variable used to explain the difficulties of integration encountered by migrants and their descendants. Lots of debates opposing the “culturalist” perspective on integration, and those stressing the structural and institutional dimensions of it, as well as mechanisms of exclusion and discrimination (Martens, 1975), developed. However, it is only in the second half the decade of 2000–2010 that the connections between arts, migration, migrants, and their descendants started to seriously catch the attention of an increasing number of European researchers in migration studies. The European research network IMISCOE [International Migration Research Network] played a pioneering role in that respect^1^ by trying to bring together migration researchers interested in arts and culture and by trying to define together news research areas.

In the initial construction of the Network of Excellence in 2004, one of the research clusters led by Steven Vertovec was labelled “Ethnic, Cultural and Religious Diversity.” The second stream of the cluster was led by Marco Martiniello and was named “Ethnic Minorities’ and Immigrants’ Cultural Productions as Forms of Political Expression”. Its first publication was a special issue of the Journal of Ethnic and Migration Studies edited by Martiniello and Lafleur (2008). It focused mainly on the links between arts, culture, and politics in the field of migration from a multidisciplinary and international perspective. It presented a state of the art of the research examining the political relevance of migrant practices in different artistic and cultural domains (music, literature, radio, clubbing, dance, visual arts, and theatre). All the contributors to the special issue were working on the links between arts, ethnicity, and migration. They found in the newly created IMISCOE cluster an opportunity to present and discuss their work and to engage in new collaborations.

In the framework of the restructured IMISCOE, the research clusters were replaced by standing committees. In 2010, POPADIVCIT [Popular Art, Diversity and Cultural Policies in Post-migration Urban Settings] was created under the auspices of Marco Martiniello, Wiebke Sievers and Ricard Zapata. The standing committee expanded as did the interest in arts and culture in the field of migration beyond IMISCOE. For example, in a book released in 2014 on immigration and the new urban landscape in New York and Amsterdam (Foner et al. (eds.), 2014), two chapters were dedicated to the impact of children of immigrants on cultural life in the two cities. Kasinitz presented his hypothesis of the “Second-Generation advantage” in New York City (2014). Sawitri Saharso with members of POPADICVIT, Christine Delhaye and Victor van de Ven (2014) showed the importance of immigrant youths for the development of urban culture in Amsterdam. In that period, more links were established with researchers working outside IMISCOE. The standing committee published two special issues (Martiniello, 2014; Zapata et al., 2017). Finally, a further restructuring of the IMISCOE Research infrastructure in 2019 led to the replacement of POPADIVCIT by DIVCULT [Superdiversity, Migration and Cultural Change]. The new standing committee is led by Marco Martiniello and Wiebke Sievers. The objectives of the new Standing Committee are to pursue and expand the work of POPADIVCIT in three ways. First, POPADIVCIT had mainly worked on arts and more specifically on music, literature, cinema and theatre. The scope of DIVCULT is broader. It includes other artistic forms and defines culture in a broader sense. This leaves space to include sports, fashion, clothing, design, food, leisure, etc. Second, the multidisciplinary character of POPADIVCIT included sociology, anthropology, cultural and literary studies, policy studies and political science. DIVCULT aims to also include economists and lawyers in the debates on superdiversity, migration, and cultural change. Third, DIVCULT also wishes to engage more actively in methodological development by continuing previous initiatives in visual studies and participatory arts-based research. In addition, the COVID-19 crisis has forced researchers in this field to develop and use on-line research methods to compensate for the impossibility to conduct traditional fieldwork during the lockdown periods.

In accordance with the objectives of DICVULT, the present article addresses two issues that are often posed when we present our work. First, it discusses why it is important to examine the links between migration, arts, and cultures. What is the additional value of such investigations to the field of ethnic and migration studies? Second, it discusses how to study those links. What are the dimensions of a fruitful methodological perspective to study the multidimensional links between arts, culture, and migration? These questions are also discussed by Wiebke Sievers in an article in this special issue. Sievers presents a different perspective and has a different disciplinary departure point.

## Why is it important to examine the links between migration, arts, and culture?

At first sight, it might seem futile to research the manifold links between migration, arts, and culture for at least two reasons. First, for the past 20 years, and even more so since the refugee reception crisis of 2015 (Rea et al., 2019), a lot of academic attention has focused on two issues. There is a focus on the growing number of migrants losing their lives in the crossing to Europe because of restrictive European migration policies. There is also growing attention for the inhuman conditions in the encampment of asylum seekers and migrants at points of entry to Europe, like Lesvos, or in the middle of Europe, like Calais. The dramatic human consequences of immigration and asylum policies have been a priority in migration research. One could say that studying the relations between arts and migration while people die at sea or freeze in camps is not only useless but may also be morally and ethically flawed. Second, most cultural, and artistic activities have been totally frozen since the beginning of the COVID-19 pandemic. It would therefore not be the appropriate moment to explore the links between arts, culture, and migration.

Despite these considerations, I claim that it is as important as ever to research the links between arts, culture, and migration. Even though the artistic and cultural world have suffered immensely since the beginning of the pandemic, artistic and cultural activities have totally stopped only for a short period of time. In a recent ethnographic study on cultural participation in five districts of the canal zone in central Brussels, it has been shown that, after a short period of total cessation, some socio-cultural workers and artists have invented new ways to pursue activities, both on-line and sometimes outdoors. These activities have proven to be very important in combatting social isolation and in fostering solidarity between the inhabitants, migrants or not, living in the research area (Mescoli & Martiniello, 2021).

Furthermore, since March 2020, the COVID-19 pandemic has largely replaced migration and asylum at the top of the media agenda and as the most prominent issue in European political debates. Migrants and refugees are among those forgotten in the management of the global health crisis, which has further increased their precariousness and, all too often, their suffering. The solidarity practices developed by parts of the local populations in European countries have been hampered by the many measures taken to fight the spread of the virus (Martiniello & Mazzola, 2020). On the one hand, migrants and refugees on European territory must try to survive with very limited rights in a state of forced immobility on the fringes of the society. On the other hand, regular access routes to Europe are still restricted, forcing many candidates for asylum and migration to attempt the near-impossible. This means they try to reach Europe by sea via increasingly dangerous routes, for instance between the coast of Senegal and the Canary Islands. Clearly, the health crisis has not put an end to either the reception crisis of migrants in Europe, or to the migratory movements whose underlying causes (social, economic, political, etc.) remain. How, in this socially and economically difficult and politically tense context, does art allow us to talk about migration and migrants? Is there an art to talking about migration? The following two case-studies drawn from my research respond concretely to these questions and demonstrate the importance of paying attention to arts in migration studies. The first presents the work and commitment of a Senegalese painter and plastic artist in Brussels. The second presents the work and commitment of an action-theatre company in the Liège region of Belgium (Martiniello, 2021).^2^

Taaw,^3^ the eldest in the Wolof language, is a Senegalese painter and plastic artist who has been living in Europe for several years. He lived in Germany and France before moving to Belgium. The son of a filmmaker and documentarian, Taaw closely follows the evolution of African societies and, in particular, the Senegalese society. We came into contact by chance, in the middle of the second lockdown in 2020. A mutual virtual acquaintance once sent me photos of about forty of Taaw’s paintings that he was trying to sell. During this period, the cultural world was once again at an almost complete standstill. The galleries were closed, and the exhibitions were interrupted, but the bills still had to be paid. Taaw, or someone close to him, thought that social networks could be used to advertise his work and maybe find some buyers. My migration sociologist eye was particularly interested in many of the superbly colourful works that I felt had a strong focus on the realities of migration. This was the origin of a strange but very rich encounter, which took place in my open garage to respect the health rules. The exchange was between two men, one a Senegalese painter and the other an Italian–Belgian sociologist, both with specific migration histories, both interested in migration and migrants, and both concerned about human rights and the glaring inequalities in the world.

We discussed the difficulties of life in these troubled times. I told him about my research in the field of migration and the multiple relationships between art and migration that I was trying to understand. I briefly presented him some of the papers, research, and collaborative projects I was developing. We also talked about his paintings, particularly those that depict an aspect of migratory realities. I remembered a short, but crucial sentence Taaw said to me during our first conversation. "I write with my painting". And in fact, Taw’s art tells the story of the often tragic, epic migration of young Senegalese migrants and travellers. Some paintings tell of the preparation for departure, others evoke the risky journey to Europe on makeshift boats. The art of Taaw speaks of migration in colour, with strength and without concessions.

At the time of our first meeting in November 2020, the press sometimes timidly reported the reopening of the clandestine maritime migration route between the Senegalese coast and the Canary Islands, the gateway to Europe. Taaw was touched in the depths of his heart by the reality of hundreds, even thousands, of deaths of young Senegalese and other Africans at sea in their quest for the European Eldorado, their mirage. For Taaw, this deadly exodus must come to an end as quickly as possible. It must be explained to the young candidates for departure that the European Eldorado does not exist and that responding to sufferings in Senegal by risking death at sea is not a good solution. As an artist and a citizen, Taaw has set himself the task of raising awareness both in Senegal and in Europe. He also considers this as an obligation of the eldest child towards his younger brothers and sisters. Furthermore, it is important to make European governments aware of the importance of putting in place win–win migration policies that would facilitate trips between African and European countries for training and work. Is this message being heard? Will it be heard? The question remains open to this day. What is clear, however, is that his paintings can be a powerful tool to raise awareness about the tragedy of migration, which he accurately depicts while also respecting human dignity.

The second case-study deals with *En Cie du Sud*,^4^ an action-theatre company that produces plays linking the history of migration with contemporary migration situations. The region of Liège in French-speaking Belgium has a migratory history linked to its industrial past, reminiscent of the mining basins of the Ruhr region in Germany and of Northern France. Unlike the latter, Liège has so far been spared from a strong presence of extreme right-wing political formations. One of the reasons for this is the existence of a dense and committed network of associations and civil society organizations. In Belgium, Liège, a mid-size superdiverse city of about 200,000 people (Vertovec, 2007), remains an antifascist bastion despite the visible impoverishment of a part of the population which is, moreover, highly diverse and multicultural.

French-speaking Belgium in general, and Liège in particular, also has a strong tradition of action-theatre, which is part of the policies of cultural democracy and permanent education promoted by the government authorities responsible for culture. Themes related to migration have been dealt with by action-theatre groups since the 1970s and 1980s. In 1996, the *Théâtre de la Renaissance de Seraing,* a city of 60.000 inhabitants adjacent to Liège, staged a show with the mysterious title, at least for spectators from outside of Liège, *Hasard, Espérance et Bonne Fortune* (Chance, Hope and Good Fortune). These are in fact the names of three collieries in the region where Italian miners arrived as early as 1946. This was the period when the Belgian and Italian governments signed a bilateral agreement for temporary migration between the two countries in exchange for a lower price on coal exported from Belgium to Italy. This agreement is still regularly referred to as the 'arms against coal deal' (Morelli, 1988). The agreement stipulated that Belgium would send coal to Italy at a competitive price in exchange for workers that would come from Italy and stay in Belgium for a limited period of 5 years. These workers extracted coal from mines that had been abandoned by Belgian workers because they were perceived as being too dangerous and because the salaries were too low.

The play, which was imagined and designed by young amateur actors, most of them of Italian origin, told the story of this migratory “adventure”, which was largely forgotten at the time. Based on precise documentary research in collaboration with historians and social scientists, the play was produced on a very original stage configuration made of a railway track. The track crosses the room and evokes the arrival of immigrant workers in railway convoys. The main roles were held by four former miners of Italian origin. They played themselves and presented a narrative and anecdotes of their migratory journey in a twirling atmosphere that went from joy to tears in an instant.

Two decades later, the troupe *En Cie du Sud* and its director, Martine De Michele, who was part of the first play as an actress, decided to revive it, updating the play while respecting the spirit of the original work. *Les fils de Hasard, Espérance et Bonne Fortune* (*The Sons of Chance, Hope and Good Fortune*) opened in autumn 2016 in Liège, 70 years after the signing of the Belgian-Italian agreement of 1946 on temporary migration. The four miners from the original play had died, so they were replaced by actors. *The Sons of Chance, Hope and Good Fortune* faithfully reproduces the approach of the first play. The strength of the testimonials remains impressive, while the link between the migration stories of the immediate post-war period and today’s migrations is very clearly established. The 2018 and 2019 editions of the play offered an exhibition for spectators to view before the performance. It included both historical documents relating to past migrations and photos of today's migrants. The troupe is also more diverse than in 1996. The actors are far from being entirely of Italian origin. A young refugee recently arrived in Belgium from Afghanistan plays the role of a young Italian immigrant in the 1950s. Other actors are Belgian, Portuguese and from Yugoslavian descent. The play evokes migration and its ruptures, reception, work, relations with local populations, life, and death. One of the final scenes of the play is particularly icy. It talks about the tragedy of Marcinelle, a colliery near Charleroi in which more than 200 miners perished in 1956, most of them Italians, due to a gas blast. The spectator, through the magic of the theatre, is led to make the link with today's tragedies in the Mediterranean Sea, in which thousands of young migrants have died.

The play was performed in Liège in 2016, 2017, 2018 and 2019 for three weeks in November of each year. The 2020 session was cancelled due to the pandemic, but the 2021 has taken take place in November. The play has also be produced in two other cities of immigration (La Louvière and Mons). So far it has been seen by more than 23,000 people. It is a moment eagerly anticipated by many each year. Because of its historical and commemorative aspects, it attracts many Italian-origin spectators from the region. However, the play is far from being an annual nostalgic rendez-vous for the Italian–Belgian community. With its profoundly contemporary elements, it attracts a public interested in the dynamics of current migrations. Finally, due to its purely theatrical and aesthetic aspects, it attracts regulars of the theatre stages who are as much interested in the professional qualities of the show as they are in its theme.

In 2018, 2019 and 2021, *En Cie du Sud* and the Centre for Ethnic and Migration Studies of the University of Liège [CEDEM] organized post-show discussions with the actors, the spectators, and researchers. The spectators could express their feelings about the play, offer their testimony, ask questions about the historical dimensions or current aspects of migration or just comment as they wished. These discussions were always very rich. Everybody felt safe to speak from her/his point of view (artistic, academic, activist, citizen, etc.). Together we showed that an informed and respectful debate on migration is still possible, even in a period often characterized by a stigmatization of migration and migrants in the media and in political arenas.

These two case-studies show that some artists have become masters in the art of talking about migration but also that scholars cannot ignore or neglect these artistic narratives on migration. Often, artists who have personally experienced the migratory process or who are descendants of migrants, draw on their personal experience and expertise to build and convey a sensitive, true and credible discourse on migration. This discourse may be easier to decipher than, for example, our scientific books and articles. Studying their work as part of migration studies is not only crucial because of the content of their expression and discourse, but also because researching the links between arts, culture and migration helps us to look at the multidimensional and complex character of migration. It highlights that migration is not only an economic, demographic, and political phenomenon, but also a cultural one. Migrants are not only muscles and arms, workers, and factors of production, but also agents of artistic and cultural change. They can have a strong social and cultural impact, both on the countries of origin and on the countries of transit and arrival. Let’s now turn to the second question addressed in this article.

## How to study the links between, arts, culture, and migration?

In previous publications (Martiniello, 2015) I suggested a general framework, constituted by five domains, to study the multiple links between arts, culture, and migration. First, at the cultural level, I suggested focusing on the ways in which artistic productions of immigrants and ethnicized, and racialized artists change the mainstream local, national, and even transnational artistic and cultural landscape*.* Second, at the social level*,* I proposed exploring to what extent artistic expressions can help build bridges between groups and facilitate *encounters* (Vertovec, 2009) between populations with different ethnic, racial and migratory origins living in the same city or the same neighbourhood. Third, at the policy level, I suggested examining how cultural institutions and cultural policies respond to the diversification of arts and culture. Are they more inclusive or exclusionary? Is there a public recognition of the cultural input of migrant and minority artists? Fourth, at the political level, I suggested examining to what extent arts and culture can be resources in political mobilization with the aim of redressing the balance of power in superdiverse countries and cities. Finally, at the economic level, the impact of immigrant and ethnic artistic expressions on the local economy also needs careful attention. In each of the five domains, several theoretical frames can be constructed and used. The aim of this paper, however, is not to discuss these theoretical constructions.

To better examine the links between arts, culture and migration at these five levels, I claim that research should be more multidisciplinary, transdisciplinary and comparative. First, it is obvious that transdisciplinary and multidisciplinary approaches are needed to encompass the five levels of analysis briefly presented above. No single academic discipline can provide enough theoretical and methodological tools to cover them all. More specifically, cultural studies, literary studies and anthropology could take the lead on the first level of investigation dedicated to cultural and artistic change linked to migration.^5^ Sociology, social psychology and again anthropology can provide valuable contributions to the study of the second level on social interactions through arts and culture. Logically one thinks of policy studies and, to a certain extent, legal studies to pave the way for examining the third level dealing with policy framing and change in the field of arts and culture. With the fourth level, political mobilization through art and culture in migration and post-migration situations, we certainly need political sociology and political science, including social movement studies. Finally, to understand the fifth level, the contribution of economic and urban geography is crucial. This list of useful academic disciplines is of course not exhaustive. There is no specific limitation to multidisciplinarity in this research field.

But beyond multidisciplinarity, I suggest that we engage in collaborative transdisciplinary research endeavours. Transdisciplinarity implies that each scholar ventures outside the comfortable borders of her/his academic discipline to learn from and understand other points of view and perspectives. This, I claim, can potentially provide additional value, and simultaneously allow us to locate our research at several of the levels described above. For example, the paper *Refugees for Refugees: Musicians between Confinement and Perspectives* (Martiniello & Sechehaye, 2019) is the result of a collaboration between me, a political sociologist of migration and ethnic relations, and Hélène Sechehaye, an ethnomusicologist. It deals with a musical project launched by a non-profit music organization, *Muziekpublique*, in Brussels, Belgium. *Muziekpublique* specializes in the promotion of so-called “world music”, a disputed category. The *Refugees for Refugees* project features traditional musicians from different countries who have found refuge in Belgium. Through this case study, we examine the complexity of elaborating a project that is based on the common identity of “refugees” while simultaneously valuing their diversity. It also discusses the impact of such participatory projects on the musicians’ careers and integration in the host country. Hélène Sechehaye was involved in the process from its onset. She was a temporary worker at *Muziekpublique* while preparing her PhD in ethnomusicology. Her input was very important. She showed that the idea that music is a universal language needs to be deconstructed, or at least be imbued with more nuance. The musicians involved in the *Refugees for Refugees* project were coming not only from different countries (Syria, Iraq, Afghanistan, Pakistan, and Tibet, etc.), but also from different musical traditions. Forming the group was an exercise in co-integrating human beings but also in creating a common musical language. Without the scholarship of Sechehaye in musicology, this aspect, which has had an impact on the career of the musicians and on the reception of their work, would have been overlooked.

Besides transdisciplinarity, comparisons can also be very useful in studying the links between art, culture, and migration. In an article dedicated to comparisons in migration studies, I discussed three different modes of comparisons: comparing people, comparing places, and comparing periods of time (Martiniello, 2013). In this specific case, I would also add the need to compare different artistic forms. Let me briefly illustrate these different modes of comparisons. First, in my article on the place of music in the political mobilization of second and third generation immigrants in Liège, I compare two multicultural groups of people: a rap crew and a group of football ultras^6^ who both use rap as a mode of political expression, identity construction and protest (Martiniello, 2020). Second, Aksoy (2020) compared the music of Alevi immigrants from Turkey in the cities of Cologne and Wuppertal in Germany. Third, it would be interesting, for example, to compare the place of arts in the political mobilization of second and third generation immigrants in the EU in the seventies and eighties with the contemporary period. The contemporary period has seen the emergence of the Black Lives Matter movement, in which arts and culture play a central role. Fourth, even though specialized artistic studies have their own merits (theatre studies, hip-hop studies, etc.), it is also interesting to compare different artistic disciplines to answer specific questions. For example, to understand the place of arts in the political mobilization of immigrants, it is more useful to compare music, theatre, literature, and painting than to focus on one specific discipline or genre. This is because the main objective is to contribute to migration studies and not so much to specialized artistic studies.

Finally, the issues of reflexivity and positionality of the researcher, as well as the construction and reproduction of categories, are as crucial in this subfield of research as they are in any other social science research (Dahinden et al., 2021). The fact that we might sometimes research artistic processes involving stigmatized and even dehumanized populations invites the researcher to be extra cautious and to pay attention to a series of important questions that are mentioned but not discussed in this article: what is the impact of the researcher’s presence in the field on the self-perception of the social actors we encounter? More generally, what is the impact of the presence of the researcher and of the research on the life of the people we interact with in the research process? To what extent is there a risk of unwillingly reproducing the stigmatizing categories often used in migration debates? How can researchers avoid speaking on behalf of the persons we interview? What impact does the research process have on the researcher’s self-definition and social position? Those questions and several others should be considered in any research project.

In the two case studies I am presenting in the article, my own history played an important role not only on the access to the field but also of the mode of collaboration both with Taaw and with the theatre troupe *En Cie du Sud.* I was born in Belgium in a family of immigrant workers from the South of Italy. My father arrived in Belgium in 1947 to work in a colliery in the Charleroi region. He ended up in a stone quarry near Liège. My mother arrived in 1950. She worked part time for a big cleaning company. Besides, she was also cleaning private homes and doing all sorts of low paid care jobs for rich families. This typical immigrant workers’ family story has no specific interest, but it is important and relevant simply because my intellectual path and career are deeply rooted in this immigrant working-class environment, materially poor but so rich in humanistic values, family love, community solidarity and socio-political engagement. In the two cases I have presented, I have discussed this personal and family migration experience with Taaw and with the theatre troupe. It helped very much to create trust between us and the reciprocal feeling that we were largely they on the same wavelength when talking about arts and migration.

## Conclusion

Migration and ethnic studies have dramatically changed over the past three decades. There has been a process of enlargement, institutionalization, professionalization, and internationalization around the study of migration and ethnic relations, and maybe also the constitution of a real multidisciplinary academic field of research. It has its own networks, journals, books series, academic degrees and so on. The range of research topics becomes broader and broader beyond the traditional pillars of migration studies, such as migration flows and migration policies, just to mention two examples. But some topics remain less developed than others. This is the case with the links between arts, culture, and migration. In this article, I have tried to explain why this topic is important and how we can try to better research it. Studying the relations between arts, culture, migration, and change is a way to acknowledge that migration is a multidimensional total social fact (Mauss, 2021). It also affects, and is affected by, arts and culture. This needs to be studied if we want to have a comprehensive understanding of the mechanisms of migration and its consequences. Furthermore, looking at migrants and their descendants as consumers and producers of artistic goods is also a way move beyond the victimising and stigmatising approaches that are very common nowadays. The lens of arts and culture acknowledges migrants’ agency. In other words, it helps to “rehumanize” migrants, who are too often “dehumanized” and reduced to statistics in current debates and policies.

## Data Availability

No applicable.

## Abbreviations

IMISCOE: International migration research network
POPADIVCIT: Popular art diversity and cultural politics in post-migration urban settings
DIVCULT: Superdiversity, migration and cultural change
CEDEM: Centre for ethnic and migration studies

## Footnotes

1 The handbook published by Dogramaci and Mersmann (2019) gives a comprehensive picture of this recent subfield of research in international migration studies.

2 This part of the article is translated and adapted from my 2021 article.

3 A press cut on Taaw: https://www.igfm.sn/between-bonn-and-dakar-pour-repandre-son-message?random

4 https://www.encompagniedusud.com/lacompagnie

5 See Wiebke Sievers’ article in this special issue.

6 Ultras as associations of football fans known for their constant passionate support.
